# Screening and identification of genetic loci involved in producing more/denser inclusion bodies in *Escherichia coli*

**DOI:** 10.1186/1475-2859-12-43

**Published:** 2013-05-02

**Authors:** Neeraj Pandey, Annapurna Sachan, Qi Chen, Kristin Ruebling-Jass, Ritu Bhalla, Kiran Kumar Panguluri, Pierre E Rouviere, Qiong Cheng

**Affiliations:** 1 BioChemical Sciences & Engineering, E.I DuPont India Pvt. Ltd. DuPont Knowledge Centre (DKC), Survey No 542/2. DS-9, ICICI Knowledge Park, Ranga Reddy District, Turkapally, Hyderabad, 500078, India; 2 BioChemical Sciences & Engineering, Experimental Station E328/B48B, Powder Mill Road and Route 141, Wilmington, DE, USA

**Keywords:** Inclusion body, Density gradient, Buoyant density, *gltA*, *glyQS*, Cell sorting, Percoll, Recombinant protein production, Downstream processing

## Abstract

**Background:**

Many proteins and peptides have been used in therapeutic or industrial applications. They are often produced in microbial production hosts by fermentation. Robust protein production in the hosts and efficient downstream purification are two critical factors that could significantly reduce cost for microbial protein production by fermentation. Producing proteins/peptides as inclusion bodies in the hosts has the potential to achieve both high titers in fermentation and cost-effective downstream purification. Manipulation of the host cells such as overexpression/deletion of certain genes could lead to producing more and/or denser inclusion bodies. However, there are limited screening methods to help to identify beneficial genetic changes rendering more protein production and/or denser inclusion bodies.

**Results:**

We report development and optimization of a simple density gradient method that can be used for distinguishing and sorting *E. coli* cells with different buoyant densities. We demonstrate utilization of the method to screen genetic libraries to identify a) expression of *glyQS* loci on plasmid that increased expression of a peptide of interest as well as the buoyant density of inclusion body producing *E. coli* cells; and b) deletion of a host *gltA* gene that increased the buoyant density of the inclusion body produced in the *E. coli* cells.

**Conclusion:**

A novel density gradient sorting method was developed to screen genetic libraries. Beneficial host genetic changes could be exploited to improve recombinant protein expression as well as downstream protein purification.

## Introduction

Bioactive peptides and proteins such as insulin [1], interferon [2,3] and erythropoietin [4] are used as curative agents in many therapeutic applications. Other peptides and proteins have found uses in a variety of industrial applications such as the pulp and paper industries and personal care industries [5]. Peptides are traditionally prepared by chemical synthesis or isolated from natural sources. Such methods are often expensive, time consuming, and characterized by a limited production capacity not scalable to industry needs. The preferred method of producing large quantities of peptides or proteins is by fermentation of recombinant microorganisms that express a gene encoding the peptide or protein of interest (POI) in the microbial host. However, recombinant microbial peptide production has a number of hurdles to overcome in order to be cost-effective. For example, peptides produced within a recombinant microbial host cell are often degraded by endogenous proteases [6], which decrease the yield and increase the cost of manufacture. Additionally, peptides production at high yields may be adversely affected by terminal heterogeneity and the amino acid composition of the peptide [6]. This is especially seen when the peptide of interest is produced in a soluble state in the production host. One of the ways to alleviate this issue is to produce the POI in an insoluble form that may accumulate within the host cell as inclusion bodies [7] thereby protecting the POI from endogenous proteases in the process. Producing the POI as inclusion bodies by fusing with an inclusion body forming tag [8] also provides a convenient means to isolate the protein from other cellular components [7] as relative high amounts of POI are enriched in the inclusion bodies. Generation of inclusion bodies at high levels in bacterial cells and the ease of purification of inclusion bodies have allowed researchers to overcome the main cost limitation and develop high throughput screens for amyloid aggregation inhibitors with potential therapeutic interest [9]. Methods have also been developed for isolation of cell-free bacterial inclusion bodies suitable for use in mammalian cell cultures and other biological interfaces [8,10]. The peptides produced as inclusion bodies could be cleaved to remove the inclusion body tag and solubilized for subsequent applications [11]. Some recent studies showed that inclusion bodies containing a high percentage of correctly folded protein are biologically active [8,12-14]. Furthermore, such inclusion bodies composed of active proteins could also be used as pure nanoparticles in diagnostics, as biocatalysts in enzyme processes, or as biopharmaceuticals [12].

One challenge associated with recombinant protein production is to manage the cost of manufacture of the desired peptide or protein of interest. Host engineering has been widely used to increase product titer and to reduce fermentation cost [15,16]. It can also be used to facilitate downstream processing of the recombinant biomass [17]. Host cell modifications that assist in enriching polypeptides comprising POIs rapidly and/or easily would decrease the cost of POI recovery and purification. However, there is very limited knowledge available to guide rational design for host engineering. For the random screening approach, it is crucial to have a relatively high throughput screening method. One of the aims of this study was to develop a screening and sorting method to selectively enrich cells bearing more/denser inclusion bodies. Another aim of the study was to demonstrate that beneficial genetic changes could be identified to improve inclusion body production and processing.

Earlier density gradient methods reported in literature served only as an analytical tool to distinguish cells producing inclusion bodies from cells not producing inclusion bodies, and it also required ultra-high speed centrifugation [18]. In this report we describe a simple density gradient centrifugation method that could not only optimally resolve cells containing inclusion bodies from those without inclusion bodies, but also cells producing different kinds and/or different amounts of inclusion bodies. We also use this method as a preparative tool to sort a BL-21 gene expression library as well as the Keio collection gene knockout library [19] transformed with plasmids expressing our inclusion body tagged peptides. We successfully enrich denser cells and identify genes/loci that result in more/denser inclusion bodies inside the cells. This is the first report of development of a novel cell sorting method for more/denser inclusion bodies based on density gradient centrifugation.

## Results

### Optimization of density gradient conditions

Density gradient conditions were optimized using percoll as the separation medium. Percoll was chosen as the density gradient medium because of its non-toxic nature, low viscosity at high concentrations, and its self-forming gradient properties. Percoll gradient was used earlier in the literature to separate cells with inclusion bodies and cells without inclusion bodies [18]. We would like to improve the sensitivity of the method not only to distinguish positives from negatives, but also to distinguish different kinds of inclusion bodies and/or different amounts of inclusion bodies inside the cells.

**Figure 1:**
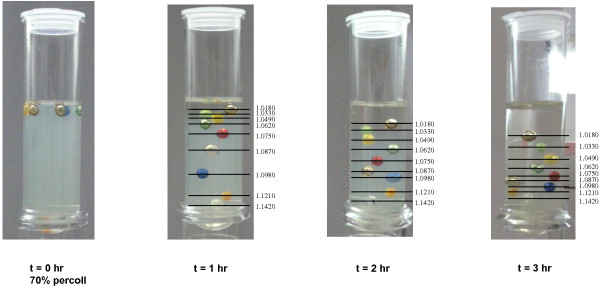
**Evaluation of 70% percoll for self-forming gradients.** t = 0 hour: before centrifuge; t = 1, 2, 3 hour: 1, 2 or 3 hours after centrifugation at 27,000 g. A set of colored density marker beads from American density materials (Staunton, VA, USA) was used as density standards to determine the range of the buoyant density formed in the tubes. The density values of the beads are shown on the right side of the tubes.

Different percentages of percoll (60%, 70%, 80%) were prepared in the centrifuge tubes and centrifuged at 27,000 g for different lengths of time (1 hr, 2 hr, 3 hr). A set of colored density marker beads (American density materials, Staunton, VA, USA) was used as density standards to determine the range of the buoyant density formed in the tubes. As shown in Figure 1, using 70% percoll and centrifuge for 1 hour at 27,000 g provides the best separation for the density range of 1.0750-1.1420, in which most *E. coli* cells fall [18].

**Figure 2:**
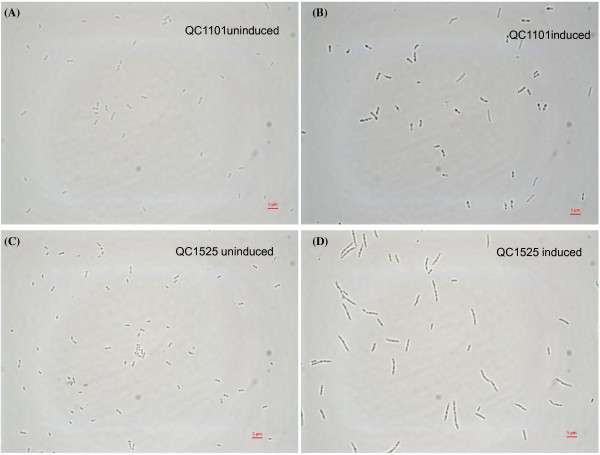
**Light microscopic pictures of cells used in the study.** Uninduced QC1101 (**A**) and induced QC1101 cells (**B**) expressing HC124 peptide; uninduced QC1525 (**C**) and induced QC1525 cells (**D**) expressing HC415 peptide. Note cells expressing HC415 inclusion bodies in panel D are distinctly larger than cells expressing HC124 inclusion bodies shown in panel B. The 5 µm scale bar is indicated.

*E. coli* QC1101 cells expressing peptide HC124 and QC1525 cells expressing a different peptide HC415 were used in this study. Both peptides were expressed as inclusion bodies by fusing with the same inclusion body tag [20]. Figure 2 shows the light microscopic pictures of the uninduced and induced QC1101 and QC1525 cells. Induced QC1525 cells appeared distinctly larger than induced QC1101 cells, and the uninduced cells looked similar size for both strains.

**Figure 3:**
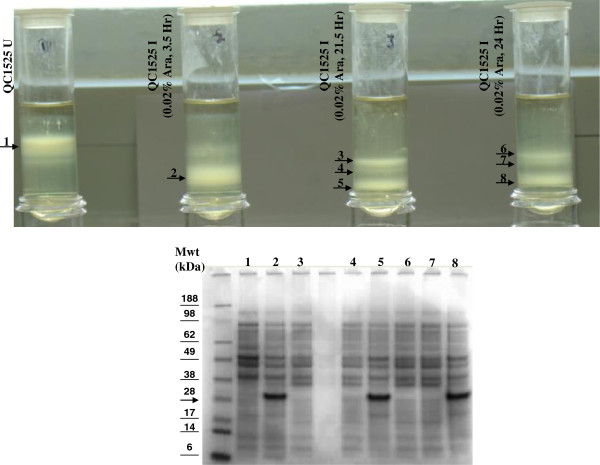
**Density gradient analysis of QC1525 cells growing under different conditions.** Top panel of the figure shows the banding patterns of cells on 70% percoll gradient. U, uninduced; I, induced. Lower panel shows SDS-PAGE analysis of the extracted bands as indicated by the arrows in the top panel. Numbers on the lanes on SDS-PAGE correspond to the numbers of the bands on density gradient. The protein band corresponding to ~28kD, where the arrow points, is the peptide of interest, which was only observed in lane 2, 5 and 8.

QC1525 cells were grown under different growth conditions, uninduced or induced with 0.02% L-arabinose for different lengths of time. The cells were mixed with 70% percoll and analyzed on the percoll gradient by centrifugation at 27,000 g for 1 hour. As shown in the top panel of Figure 3, the uninduced cells and the induced cells of QC1525 can be well separated on the density gradient. Induction for longer periods of time yielded heavier cells likely due to more inclusion bodies produced inside the cells. The cells that formed the distinct bands on the density gradient were pipetted out into separate tubes. They were lysed and their protein profiles were examined on the SDS-PAGE (Figure 3 bottom panel). The uninduced cells showed the *E. coli* background proteins. The heavy bands (2, 5, 8) in each of the induced cultures contained the expressed peptide of interest around 28 kD. The lighter bands in any of the induced cultures did not contain the expressed POI. This confirmed that cells expressed POI as inclusion bodies were heavier than cells did not express POI, and their buoyant density differences allowed them to be well separated on density gradient. Even in the induced cultures, there was a subpopulation of cells that appeared to be uninduced for POI expression.

**Figure 4:**
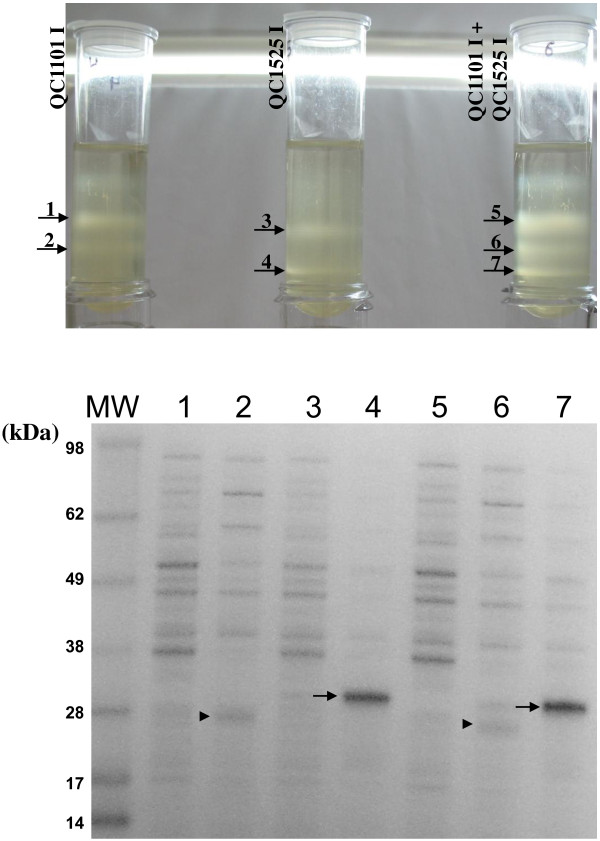
**Density gradient analysis of mixed cultures of induced QC1101 and QC1525.** Top panel of the figure shows the banding patterns of individual cultures and the mixed cultures on 70% percoll density gradient. I, induced. Arrows indicate the bands that were extracted for SDS-PAGE analysis. Lower panel shows the SDS-PAGE analysis of the extracted bands. Numbers on the lanes on SDS-PAGE correspond to the numbers of bands on density gradient. Arrowheads show HC124 peptide, while arrows show HC415 peptide in the SDS-PAGE gel.

In order to test if cells producing different POIs as inclusion bodies could be separated on the percoll density gradient, induced QC1101 and induced QC1525 cultures were analyzed on the 70% percoll gradient either as separate cultures or as mixed cultures. Figure 4 showed that QC1101 induced cultures formed distinct bands on the density gradient. Band 1 was confirmed to be uninduced cell population without POI expression and band 2 contained induced cells with HC124 expression (Figure 4, lower panel, shown by arrowhead). Similarly in the induced QC1525 cultures, band 3 contained uninduced cell population without POI expression and band 4 contained induced cells with HC415 expression (Figure 4, lower panel, shown by arrow). When induced QC1101 culture and induced QC1525 culture were mixed, the banding pattern of the mixed culture looked like the combination of the banding patterns of the individual cultures on the density gradient. The buoyant densities of the uninduced population in both cultures (band 1 and band 3) were similar and formed band 5 in the mixed culture. The buoyant densities of the induced population in both cultures (band 2 and band 4) were quite different and formed distinct bands (band 6 and band 7) in the mixed cultures. Band 6 was confirmed to contain induced cells expressing HC124 (Figure 4, lower panel, shown by arrowhead) and band 7 was confirmed to contain induced cells expressing HC415 (Figure 4, lower panel, shown by arrow). The cells expressing two different POIs could be clearly separated on density gradient. In this case induced QC1525 cells were heavier on density gradient, which seemed to be consistent with microscopic observation of larger QC1525 cells packing more inclusion bodies (Figure 2) and more HC415 expression on SDS-PAGE.

### Enrichment of denser cells from a gene expression library by density gradient sorting

After establishing the density gradient method to separate cells based on their inclusion body content, we explored to see if we could use this method to sort libraries to identify genetic traits that may cause an increase of the buoyant density of the inclusion bodies. A plasmid expression library RK1 was constructed in the QC1101 host using ~2-3 Kb fragments from BL21 genomic DNA. QC1101 cells produced HC124 peptide as the POI. The library was grown to OD_600_ ~0.6 and induced with 0.02% L-arabinose for 5 hours. Cells were then separated in the percoll density gradient and the heaviest portion of cells was collected. These cells were grown up again and the density gradient sorting process was repeated. Cells were sorted in this manner five times and at every cycle an aliquot of the sorted cells were plated out on LB plates containing ampicillin and kanamycin. Approximately twenty colonies were randomly picked for plasmid preparation and sequenced with forward and reverse primers. Obtained sequences of each sort were used for BLAST search [21] to identify genes represented in the population.

**Figure 5:**
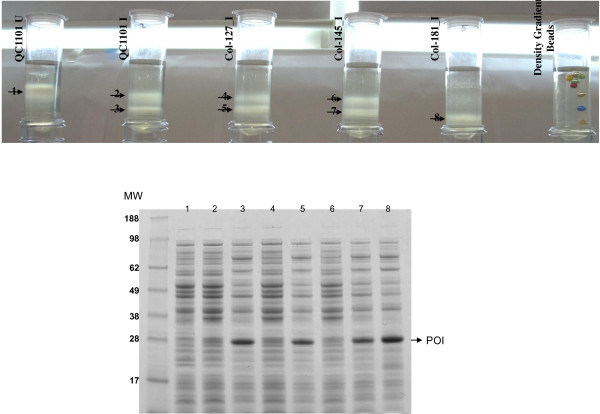
**Density gradient analysis of isolates of interest.** U, uninduced; I, induced. Notice increase in buoyant density and population of induced cells obtained from colony 181 (top panel). The induced cells from colony 181 also show higher percentage of POI production (bottom panel, lane 8) compared to the QC1101 induced cell control (lane 3). The numbers on the lanes on SDS-PAGE correspond to the numbers of the bands on density gradient.

A gradual increase of *ysaB*-*glyQ*-*glyS* locus was observed in the sorted population. After sort 1 approximately 11% of the sequenced clones contained this locus, and after sort 2 approximately 8% of the clones had this locus. By sort 3, 59.1% of the sequenced clones were enriched for this particular locus. Sort 4 and Sort 5 had 80.9% and 90.5% enrichment respectively of this particular gene region. This demonstrated that repeated density gradient sorting led to the increased representation of certain regions of *E. coli* genome, implying that certain genes in the library of the BL21 genome may play a role in affecting inclusion body density. Having obtained a list of genes by density gradient screening of cells in the RK1 library, we decided to test the individual isolates for differences in buoyant density compared to the control. The control QC1101 strain contained the HC124 expression plasmid. The isolates contained the same HC124 expression plasmid and another plasmid with a BL21 genomic fragment. The isolates and the control were grown and induced for 5 hours. The individual isolates were first tested in micro-density gradient to examine if their density pattern differed from induced QC1101 (data not shown). Clones of interest identified by micro-density gradient were subsequently tested on large scale density gradient and expression of POI was also compared between identified clones and induced QC1101 control (Figure 5). Figure 5 demonstrates that in the QC1101 control, no band corresponding to POI was observed without induction (lower panel, lane 1), and upon induction cells (upper panel, band 3) expressing HC124 peptide of interest (lower panel, lane 3) were observed. Most isolates bearing a genomic fragment from BL21 showed similar profile as the control, which indicated that those genomic fragments had no effect on expression of HC124 peptide. However, colony number 181 consistently demonstrated heavier buoyant density both in micro and macro density gradient runs. It is also interesting to note that the induced cell population in Colony 181 sample was the predominant population and the uninduced cell population was a minor population, comparing to control cells which had about equal amount of uninduced and induced population. When the induced cells from colony 181 (band 8) were extracted and analyzed on SDS-PAGE, it showed approximately 33 percent increase of POI production as quantified by densitometry (lane 8) comparing to the control (lane 3). Sequencing of this clone revealed that the insert spanned from 3585956 to 3587860 of the BL21 genome and contained full length *ysaB*, *glyQ* and partial *glyS* genes. The *glyQ* and *glyS* genes encode glycine tRNA synthetase alpha and beta subunit respectively [22,23]. The *ysaB* gene encodes a predicted protein with unknown function. As in *E. coli* there is one glycine tRNA synthetase for charging of glycine tRNA with its cognate glycine amino acid [24,25], overexpression of the *glyQS* genes on plasmid might increase the availability of glycine tRNA synthetase. The HC124 peptide expressed in QC1101 has a glycine content of 23%, which is about twice as high as mol% of glycine in *E. coli* host proteins [26]. It is likely that preponderance of *ysa*B-*glyQ*-*glyS* region in identified clones might be relating to the higher glycine content in the peptide of interest HC124. Retransformation of the plasmid from colony 181 into the same fresh host showed similar phenotype of heavier cells on density gradient and more protein expression on SDS-PAGE, suggesting that the plasmid containing the *ysa*B-*glyQ*-*glyS* fragment from BL-21 in colony 181 was responsible for this phenotype.

### Enrichment of denser cells from a gene knockout library by density gradient sorting

The Keio collection [19] is a collection of ~4000 in frame, single gene deletion strains of *Escherichia coli* K12 in a common strain background of BW25113. The QC3800 library is consisted of the mixture of cells with individual gene deletion in the Keio collection expressing the same inclusion body forming peptide HC415. The QC3800 library was subject to three rounds of density gradient sorting of denser cells in 70% percoll gradient. Sequencing of 96 randomly picked colonies from the last round of sorting identified several gene deletion strains enriched in the sorted population. The G1, B3 and C6 isolates each contain deletion in the *cspA, cspC* and *cspE* gene, respectively. These all belong to the cold shock family of proteins that were shown to be involved in a variety of cellular processes. The *cspA* and *cspC* encodes the same family of DNA binding transcriptional activators that are involved in stress response [27,28]. The *cspE* encodes a transcriptional antiterminator that is involved in regulation of RNA stability [29]. They are non-adjacent to each other and located in different regions in the *E. coli* chromosome. The F1 and A12 isolates both contain deletion in the *gltA* gene, which encodes the citrate synthase [30]. The B11, D2 and E4 each contain deletion of an unknown function gene *ydcP*, *ygcN* and *yhaB* identified twice.

**Figure 6:**
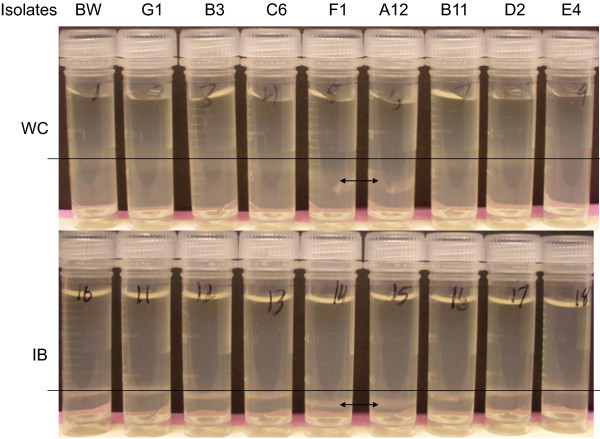
**Microdensity gradient centrifugation of selected isolates from density gradient sorting of the QC3800 library.** Top panel is the whole cells (WC) and bottom panel is the inclusion bodies (IB). The control BW25113 containing the expression plasmid is labeled BW. The density positions of the controls are indicated by the straight lines. The density positions of isolates F1 and A12 are indicated by the arrows.

The eight individual isolates (G1, B3, C6, F1, A12, B11, D2, and E4) with multiple hits were selected for density gradient analysis in microfuge tubes. In Figure 6, the top panel shows the whole cell (WC) samples which had broader density gradient bands. The crude inclusion bodies were also prepared as described in the Methods section. The lower panel shows the more discrete density gradient bands of the corresponding crude inclusion body (IB) samples. For both the whole cell bands and the IB bands, isolates F1 and A12 showed increased buoyant densities when compared to the respective control samples from BW25113 containing the same peptide expression plasmid (Figure 6, BW). The increased buoyant densities of the two positive hits were also confirmed on 70% percoll gradient in the 30-mL large centrifuge tubes.

**Figure 7:**
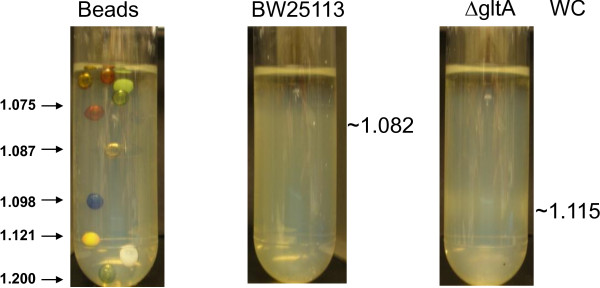
**Confirmation of increased buoyant density in Δ*gltA.*** Density gradient analysis of the whole cells producing the inclusion bodies (top panel WC) and the crude inclusion bodies (bottom panel IB) from the parent strain BW25113 and the JW0710 strain with Δ*gltA.* Density standard beads of various buoyant densities were separated under the same conditions

To confirm that the *gltA* deletion of the host strain in F1 and A12 isolates caused the increased buoyant density of the inclusion bodies and the cells producing the inclusion bodies, the peptide expression plasmid was transformed into the JW0710 strain in the Keio collection containing the *gltA* deletion. Duplicates of the cultures and the control were grown and induced as described in the Methods section. Figure 7 shows the whole cells (top panel) and the crude inclusion bodies (bottom panel) from these cultures analyzed by density gradient centrifugation. A set of density marker beads was used as density standards to determine the buoyant density values for this experiment. The buoyant density of the control cells had a wider distribution of about 1.08-1.09. The buoyant density of the Δ*gltA* cells was estimated to be about 1.115. The buoyant density of the inclusion bodies from the control strain was approximately 1.121 and the buoyant density of the inclusion bodies from the Δ*gltA* cells was approximately 1.142. Results confirmed that the cells and the inclusion bodies from the Δ*gltA* strain showed increased buoyant densities than those from the control strain. JW0709 strain containing the adjacent *ybgD* deletion was also tested and Δ*ybgD* did not have any effect on the buoyant densities of the inclusion bodies or the cells (data not shown).

It is not clear why the host Δ*gltA* would increase the buoyant density of the inclusion bodies produced in the cells. SDS-PAGE analysis showed similar amount of peptide was produced in the Δ*gltA* strain (data not shown). It is likely that Δ*gltA* affected the packing of the peptide in the inclusion bodies, which resulted in denser inclusion bodies produced in the Δ*gltA* cells.

## Discussion

Robust protein production in microbial hosts and efficient downstream purification are two critical factors that could significantly reduce cost for microbial protein production by fermentation. A simple and convenient screen that allows identification and isolation of beneficial variants from libraries of tens of thousands of variants is crucial to guide metabolic engineering to improve protein production and processing. Cells with distinct shapes and/or intracellular contents are generally distinguished and sorted by fluorescence activated cell sorting (FACS) either without labelling (forward scattering and side scattering) or with fluorescence labeling [20,31]. However, FACS requires the expensive cell sorter, and the FACS sensitivity for bacterial cells is usually not very high. Inclusion body profiles can be distinguished under a microscope, but to sort cells based on inclusion body profiles quantitated by physical techniques such as impedance measurements is complex and time consuming [32]. This paper describes the first report of development of a novel cell sorting method for more/denser inclusion bodies based on density gradient centrifugation. This method was successfully applied to identify host genes/loci, *ysaB-glyQ-glyS* from an expression library and Δ*gltA* from a knockout library that resulted in cells with higher buoyant densities. In the *glyQS* expression clone, inclusion body peptide production increased 33%. In the *gltA* deletion strain, the inclusion body production amount did not increase, even though the density of inclusion bodies and inclusion body containing cells increased.

It was described many years ago in a GFP fluorescence study that expression from the *araBAD* promoter resulted in mixed population of induced and uninduced cells at subsaturating inducer concentrations [33]. We used saturating concentrations of L-arabinose (0.02% or 0.2%) for induction and still observed the mixed population on density gradient. The ratio of induced versus uninduced cells appeared to be different for different peptides. The uninduced cells were collected and regrown in ampicillin medium with L-arabinose induction. The mixed population pattern was observed again on density gradient. This suggested that the uninduced cells were not simply due to loss of the expression plasmid. The induced cells were also collected and regrown similarly, and the same pattern of mixed population was observed. Increase of the inducer concentration did not shift all cells to induced population. This implied that there might be a bottleneck for protein expression in the cell. If the bottleneck was relieved, the ratio of the induced cells might be increased as in the clone that contained the *glyQS* loci (Figure 5) and lead to increase of peptide production. The density gradient analysis is a useful tool that could be used to help to identify and relieve production bottlenecks for increased protein production.

To improve fermentation productivity and reduce fermentation cost, it is important to optimize fermentation conditions such as the timing of induction, inducer concentration and length of fermentation for production of each peptide/protein. SDS-PAGE analysis could show total protein production in the whole cell population. The density gradient analysis could dissect more to monitor induced and uninduced populations separately and determine specific cell productivity in the induced cells. Having the in-depth understanding of microbial cell factories would guide metabolic engineering to increase protein production. The density gradient analysis might also help to diagnose issues such as loss of plasmids or cell lysis during fermentation.

In the *gltA* deletion strain, no increase of inclusion body production was observed even the density of inclusion bodies and inclusion body containing cells increased. It is likely that the increased density in this case might be due to denser packing of inclusion bodies or possible interaction of inclusion bodies with percoll. Measuring settling velocity of inclusion bodies in a matrix free system may shed more light. Nevertheless, denser inclusion bodies and inclusion body containing cells are beneficial for recovery and purification in downstream processing. Downstream processing steps involve cell harvesting by centrifugation, homogenization of the cells to release the inclusion body peptides into the supernatant, and recover and wash inclusion body peptides by centrifugation. Denser inclusion bodies and inclusion body containing cells could reduce centrifugation speed/time and increase sample processing throughput especially for large scale continuous centrifugations frequently used in downstream processing.

## Conclusions

In this paper we described a simple method of density gradient centrifugation that can be used as a tool to improve recombinant protein production. The method can be used not only to monitor induced cell population, but also to sort and selectively enrich more/denser inclusion body containing cells. This method was successfully applied to identify host genes/loci, *ysaB-glyQ-glyS* from an expression library and Δ*gltA* from a knockout library that resulted in cells with higher buoyant densities. In the *glyQS* expression clone, inclusion body peptide production increased 33%. In the *gltA* deletion strain, the inclusion body production amount did not increase, even though the density of inclusion bodies and inclusion body containing cells increased. The density gradient centrifugation method can be used to improve protein production at different stages, such as to identify production bottlenecks in microbial cell factories, to optimize induction and fermentation conditions, and to facilitate downstream processing.

## Methods

### Peptides, plasmids and strains

Peptides, plasmids and strains used in this study are listed in Table 1. Briefly, two different peptides of interest (POI), HC124 and HC415, were used in this study, and the amino acid sequences were shown in Table 1. Each POI was fused with a same inclusion body tag (QQRFQWQFEQQPRGQQRFQWQFEQQPRGQQRFQWQFEQQPEGQQRFQWQFEQQ) and expressed by the *araBAD* promoter on the pBR vector. HC124 expressed in the QC1100 (MG1655 ΔaraBAD ΔslyD) host resulting QC1101 strain. HC415 expressed in the QC1100 host resulting QC1525 strain.

**Table 1:** Peptides, plasmid and strains used in the study

**Name**	**Characteristics**
HC124 (POI)	gsdpgipwwniraplnagagipwwniraplnaggsgpgsggntsqlstgggntsqlstggpkkpgdpgipwwniraplnagagipwwniraplnaggsgpgsggntsqlstgggntsqlstggpkkpgd
HC415 (POI)	gsdpsaqsqlpdkhsglherapqrygpeeaakkeeaakkpahinktnphqgnhhsektqrqgsggggsgsggggsdshhnhhkqdsrpqhrktpngggdshhnhhkqdsrpqhrktpngk
QC1100	MG1655 Δ*araBAD* Δ*slyD*, expression host for POI
QC1101	QC1100 host expressing HC124 under pBAD promoter on pBR vector
QC1525	QC1100 host expressing HC415 under pBAD promoter on pBR vector
pDCQ601	Vector containing a linker of multiple cloning sites introduced at the *EcoR*I site downstream of the Pcat promoter on pBHR1 (MoBiTec, Germany)
RK1 library	BL21 genomic DNA fragments expressed in QC1101 producing HC124
QC3800 library	Mixture of Keio collection strains producing HC415
BW25113	Parental strain for the Keio collection
JW0710	BW25113 Δ*gltA*, Keio collection strain containing Δ*gltA* knockout
Colony 181	Isolate from RK1 library that contained the *ysaB*-*glyQ*-*glyS* locus

QC3800 knockout library was constructed by pooling the individual Keio collection knockout strain and transforming HC415 expression plasmid into the pooled cells. BW25113 was the parental strain of the Keio collection. RK1 expression library was constructed as described in the following section.

### Construction of BL-21 genomic DNA expression library

To identify genes that can increase the buoyant density of *E. coli* cells, genomic DNA from *E. coli* BL21 strain was extracted and an expression library was constructed. Genomic DNA fragments of ~ 2 to 3 kb in size were obtained from *E. coli* BL21 strain by partial digestion of the BL21 genomic DNA with *Tas* I restriction enzyme at a non-ideal temperature (to prevent complete digestion) of 55°C for 50 minutes. The digested fragments were subjected to agarose gel electrophoresis alongside a DNA molecular weight ladder. Agarose slices containing genomic DNA fragments corresponding in size to approximately 2 to 3 kb were excised from gel and the DNA therein eluted.

Vector pDCQ601 was digested with 5 units of *Eco*RI (New England Biolabs, USA) at 37° overnight. The 5.3 kb fragment corresponding to *Eco*RI digested plasmid was eluted from the agarose gel slice. The eluted *Eco*RI digested plasmid was treated with Antarctic phosphatase (New England Biolabs, USA) for 1 hour at 37°C, followed by heat inactivation of the Antarctic phosphatase by incubating the reaction mix at 65°C for 5 minutes. The reaction mix was cleaned by using DNA Cleanup Kit (Zymo Research, USA). Aliquots of the eluates of both *Eco*RI digested and phosphatase treated vector and the *Tas* I digested genomic DNA (2–3 Kb fragments) were combined for ligation using Roche Rapid DNA Ligation Kit (Roche, USA). The ligation reaction used vector ends and insert ends ratio of 1:3 at 24°C for 15 min in a total volume of 21 µL. An aliquot of 10 µL of the ligation mix was used to transform CB5 alpha competent cells (Chromous Biotech, Bangalore, India; Catalog # PCR 16- NP) which were then plated on LB with kanamycin 50 µg/ml plates. An average of 2000 colonies per plate was obtained. Additional ligations were done in four batches and four plasmid pools of approximately 15000 colonies each were prepared and transformed into QC1101 electrocompetent cells to have ~ 10-fold coverage of the *E. coli* BL21 genome. Thirty colonies from transformed QC1101 cells were randomly picked and tested by colony PCR. All colonies were found to have ~2-3 kb inserts. Sequencing of more than 30 plasmids isolated from these colonies revealed that all of them had unique DNA inserts. The BL21 genomic DNA library with 2–3 kb insert size (designated as RK1 library) was used to screen for genes that may increase the buoyant density of *E. coli* cells synthesizing polypeptides that accumulate in the form of inclusion bodies.

### Growth and induction conditions

To produce the recombinant protein in the cells, a single colony of appropriately transformed *E. coli* was inoculated in 3 ml of Luria-Bertani (LB) medium containing ampicillin (100 µg/ml) for overnight growth. Next morning, 1-2% of this inoculum was added to 250 ml flask containing 50 ml of LB-carbenicillin (100 µg/ml) medium or LB-carbenicillin-kanamycin (50 µg/ml) medium as appropriate and the cultures were shaking at 250 rpm at 37°C. When the optical density values (OD_600_) of the cultures reached ~0.6, the *E. coli* cells were induced with 0.02% or 0.2% L-arabinose and incubated for another 5 hours or 22 hours as indicated in the experiment, OD_600_ of the cells following incubation were recorded. An aliquot of cells were pelleted in eppendorf tubes and stored at −20°C for protein analysis, and the rest of cells were used for density gradient centrifugation.

### SDS-PAGE Analysis

For SDS PAGE analysis of the sorted cells, bands of interest were extracted following density gradient centrifugation. All bands were aspirated out with sterile 10 ml syringes or 1 ml pipet tips. The cells were washed with 1 X PBS by centrifuging at 5500 g at 4°C for 15 min and OD_600_ was taken. OD_600_ of the extracted cells were normalized, and cells were lysed by heating to 100°C for 15 mins in a heated dry block and centrifuged briefly. The samples were run on a NuPAGE® 4-12% Bis-Tris gel with the SeeBlue® Plus2 pre-Stained standard (Invitrogen, Carlsbad, USA). At the end of the run the gel was rinsed with water, stained with SimplyBlue™ (Invitrogen, Carlsbad, USA) and destained with deionized water. The gel was subsequently scanned in GS-800 calibrated™ densitometer (Bio-Rad laboratories, USA) and bands quantitated by Quantity One® (Bio-Rad laboratories, USA) software.

### Inclusion body isolation

Whole cells were frozen at −80°C and subsequently lysed using CelLytic Express (Sigma-Aldrich, St Louis, MO) for 1 hour at 37°C. The crude inclusion bodies were pelleted from the lysed cells with centrifugation at 14,000 rpm for 5 minutes, then resuspended in water and loaded in 70% percoll. The buoyant density of the crude inclusion bodies was analyzed similarly by density gradient centrifugation.

### Density gradient centrifugation and sorting

Induced *E. coli* cells of total OD_600_ of 9 were mixed with final concentrations of 150 mM NaCl and 70% Percoll, and the final volume was adjusted to 30 ml with sterile LB. All of the components were mixed in 50 ml screw capped tubes by inverting 10 times and then transferred to polycarbonate centrifuge tubes with snap on lids. The cells and solutions used could also be scaled down proportionally in 2 ml screw capped microcentrifuge tubes for microdensity gradient analysis. The tubes were then spun at 27000 g at 20°C for a period of time ranging from 1–3 hours, following which the centrifugation was gradually brought to halt by keeping deceleration value of 1 in Sorvall super speed centrifuge. Density marker beads were loaded in a separate tube in 70% percoll and were centrifuged under the same conditions. The centrifuged samples were then photographed by placing them in front of source of light for better visualization of gradient bands.

To collect cells, the visible bands were aspirated out using separate sterile syringes or 1 ml pipet tips inside the sterile hood. The aspirated bands containing cells had a volume ranging from 2–3 ml. The collected cells were washed with 10 volumes of sterile 1X PBS. For repeated sorting, the cells were inoculated into fresh medium for another round of growth, induction and density gradient centrifugation. At the end of enrichment, the recovered cells were diluted and plated on LB plates with appropriate antibiotics. Colonies were randomly picked for sequencing to identify the genetic change in each strain. The individual isolates and the control strain were also grown and induced as described earlier. The buoyant density of the inclusion body producing cells was analyzed by density gradient centrifugation. In addition, the crude inclusion body preparations obtained from the lysed cells were loaded in 70% percoll, and the buoyant density of the crude inclusion bodies was also analyzed similarly by density gradient centrifugation.

## Abbreviations

E. coli: *Escherichia coli*; POI: Peptide of interest; or protein of interest; WC: Whole cell; IB: Inclusion body; FACS: Fluorescence activated cell sorting; LB: Luria Bertani; OD600: Optical density at 600 nm; PBS: Phosphate buffered saline; SDS-PAGE: Sodium dodecyl sulfate polyacrylamide gel electrophoresis; GFP: Green fluorescence protein.

## Competing interests

The authors declare that they have no competing interests.

## Authors’ contributions

NP crafted the study design, performed the experiments, did sequence alignments and wrote the draft of the manuscript. AS carried out the molecular genetic studies, performed the experiments and was involved in the drafting the manuscript. RB and KKP were involved in generation of BL-21 genomic DNA library. QC (Qi Chen) performed the density gradient sorting of the gene knockout library. KRJ participated in the confirmation of the individual isolates. PER participated in the experiment design and data interpretation. QC (Qiong Cheng) conceived of the study, and participated in its design and coordination and helped to write and edit the manuscript. All authors read and approved the final manuscript.
